# Activin A/BMP2 chimera AB235 drives efficient redifferentiation of long term cultured autologous chondrocytes

**DOI:** 10.1038/srep16400

**Published:** 2015-11-13

**Authors:** G. Jiménez, E. López-Ruiz, W. Kwiatkowski, E. Montañez, F. Arrebola, E. Carrillo, P. C. Gray, J. C. Izpisua Belmonte, S. Choe, M. Perán, J. A. Marchal

**Affiliations:** 1 Biopathology and Regenerative Medicine Institute (IBIMER), Centre for Biomedical Research, University of Granada, Granada E-18100, Spain; 2 Department of Human Anatomy and Embryology, Faculty of Medicine, University of Granada, Granada E-18012, Spain; 3 Biosanitary Institute of Granada (ibs.GRANADA), University Hospitals of Granada-Univesity of Granada, Granada, Spain; 4 Department of Health Sciences, University of Jaén, Jaén E-23071, Spain; 5 Structural Biology Laboratory, Salk Institute for Biological Studies, La Jolla CA 92037, California, USA; 6 Department of Orthopedic Surgery and Traumatology, Virgen de la Victoria University Hospital, Málaga, Spain; 7 Department of Histology, Faculty of Medicine, University of Granada, E-18071 Granada, Spain; 8 Clayton Foundation Laboratories for Peptide Biology, Salk Institute for Biological Studies, La Jolla CA 92037, California, USA; 9 Gene Expression Laboratory, Salk Institute for Biological Studies, La Jolla CA 92037, California, USA; 10 Qualcomm Institute, Univ. California, San Diego, La Jolla, CA 92037, USA

## Abstract

Autologous chondrocyte implantation (ACI) depends on the quality and quantity of implanted cells and is hindered by the fact that chondrocytes cultured for long periods of time undergo dedifferentiation. Here we have developed a reproducible and efficient chondrogenic protocol to redifferentiate chondrocytes isolated from osteoarthritis (OA) patients. We used morphological, histological and immunological analysis together with a RT-PCR detection of collagen I and collagen II gene expression to show that chondrocytes isolated from articular cartilage biopsies of patients and subjected to long-term culture undergo dedifferentiation and that these cells can be redifferentiated following treatment with the chimeric Activin A/BMP2 ligand AB235. Examination of AB235-treated cell pellets in both *in vitro* and *in vivo* experiments revealed that redifferentiated chondrocytes synthesized a cartilage-specific extracellular matrix (ECM), primarily consisting of vertically-orientated collagen fibres and cartilage-specific proteoglycans. AB235-treated cell pellets also integrated into the surrounding subcutaneous tissue following transplantation in mice as demonstrated by their dramatic increase in size while non-treated control pellets disintegrated upon transplantation. Thus, our findings describe an effective protocol for the promotion of redifferentiation of autologous chondrocytes obtained from OA patients and the formation of a cartilage-like ECM that can integrate into the surrounding tissue *in vivo*.

The high incidence of chondral lesions and the lack of a definitive treatment, particularly due to the intrinsic characteristics of the cartilage tissue, have an important impact on the health services systems in developed countries. Among approaches to treat articular cartilage lesions, autologous chondrocyte implantation (ACI) has been established as a good clinical therapeutic strategy for treating small injuries^1^. Nevertheless, this procedure has important limitations including the restricted number of chondrocytes that can be isolated from a patient biopsy. In fact, in order to increase the number of cells, freshly isolated chondrocytes are cultured and expanded *in vitro*. However, this leads to the problem of loss of the chondrocyte phenotype due to cell dedifferentiation that occurs during prolonged monolayer culture^2^. This dedifferentiation causes chondrocytes to lose their round shape and become flattened fibroblast-like cells with an increased proliferative capacity and is accompanied by changes in gene expression and surface markers including decreased Col II and aggrecan and increased levels of Col I, Col X and COMP^3^. This loss of the differentiated chondrocyte phenotype upon culture *in vitro* represents a major disadvantage of the ACI technique because a decreased ratio of collagen type II/I results in production of an extracellular matrix typical of fibrotic tissue that might compromise cartilage regeneration^4^.

Since the success of ACI depends on the number and quality of the cells to be implanted into the chondral lesion, approaches to revert dedifferentiation, called redifferentiation, are being investigated. In this respect, some studies have focused on using 3D cultures^5^ or growth factors, such as members of the TGF-β superfamily including bone morphogenetic proteins (BMPs)^6^,^7^.

BMPs and activins are structurally related members of the TGF-β superfamily of ligands but signal through different pairs of receptors^8^. Activin A exhibits very high affinity for its type II receptors, ActRII and ActRIIB, whereas BMP2 possesses low affinity for these receptors and higher affinity for its type I receptors. Since Activin A and BMP2 bind different type I receptors they activate distinct signalling pathways, i.e. Activin A activates SMAD2/3 transcription factors while BMP2 activates SMAD1/5/8 transcription factors^9^. We previously reported the creation of chimeric ligands based on systematic swapping of BMP2 and Activin-A sequences using a strategy termed Random Assembly of Segmental Chimera and Heteromers (RASCH)^10^. We found that one of these chimeras, AB235, significantly promotes chondrogenic differentiation of adipose-derived stem cells^11^.

Here we demonstrate that AB235 effectively induces redifferentiation of functional osteoarthritis (OA) patient-derived dediferentiated chondrocytes. Our results establish a novel protocol for re-establishing and maintaining the mature chondrocyte phenotype when cells are cultured for extended periods *in vitro*. This approach has the potential to facilitate and enhance treatment of cartilage-related injuries using ACI.

## Results

### Chondrocyte dedifferentiation upon monolayer culture

**Figure 1:**
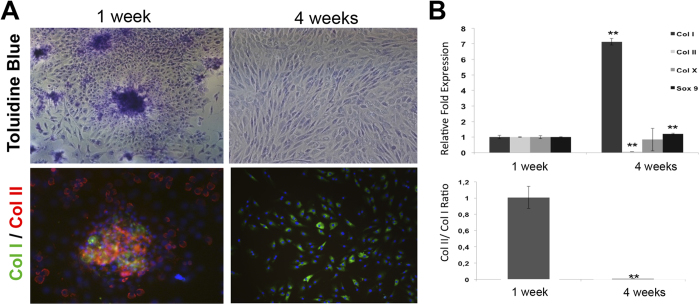
Dedifferentiation of chondrocyes grown in monolayer culture. (**A**) Chondrocytes cultured for 1 week or 4 weeks were stained with Toluidine Blue and immunolabeled for Col I (green) and Col II (red). (**B**) Real-time PCR analysis of selected chondrogenic markers after 4 weeks of monolayer cell culture. The bottom graphic shows the ratio of Col II versus Col I expression during the process of differentiation. **Statistical significance indicated (p < 0.01).

Freshly isolated chondrocytes were grown in monolayer culture up to passage 6 (P6) to ensure a complete dedifferentiation. Dedifferentiation was evident as soon as P3, when the proliferation of the cells increased and some chondrocytes started to change morphology and adopt a spindle-like fibroblastic shape. After 4 weeks all cells had increased in size and adopted a fibroblast-like appearance Fig. 1(A), while control chondrocytes cultured for only 7 days retained a typical polygonal and star-shaped morphology. Toluidine Blue staining, which reflects synthesis of glycosaminoglycans (GAGs), was clearly decreased in cell monolayers at P6 relative to control chondrocytes Fig. 1(A). Further, chondrocytes cultured for 4 weeks showed decreased Col II expression (red staining) and increased Col I expression (green staining) while control chondrocytes showed the opposite Fig. 1(A).

Finally, we evaluated the expression of selected chondrogenic markers by qRT-PCR Fig. 1(B) and found that chondrocytes at P6 have increased expression of Col I (p < 0.01) and Col X and decreased expression of Col II (p < 0.01) when compared with control cells Fig. 1(B). A slight increase of Sox 9 expression (p < 0.01) was also detected specifically in long-term cultured chondrocytes. Furthermore, a significant decrease of the Col II/Col I ratio (p < 0.01) was observed in chondrocytes at P6 when compared to the ratio in the control cells. Together, these results indicate chondrocytes cultured in monolayer for four weeks adopt a dedifferentiated, fibroblast-like phenotype.

### AB235 promotes redifferentiation of dedifferentiated chondrocytes

**Figure 2:**
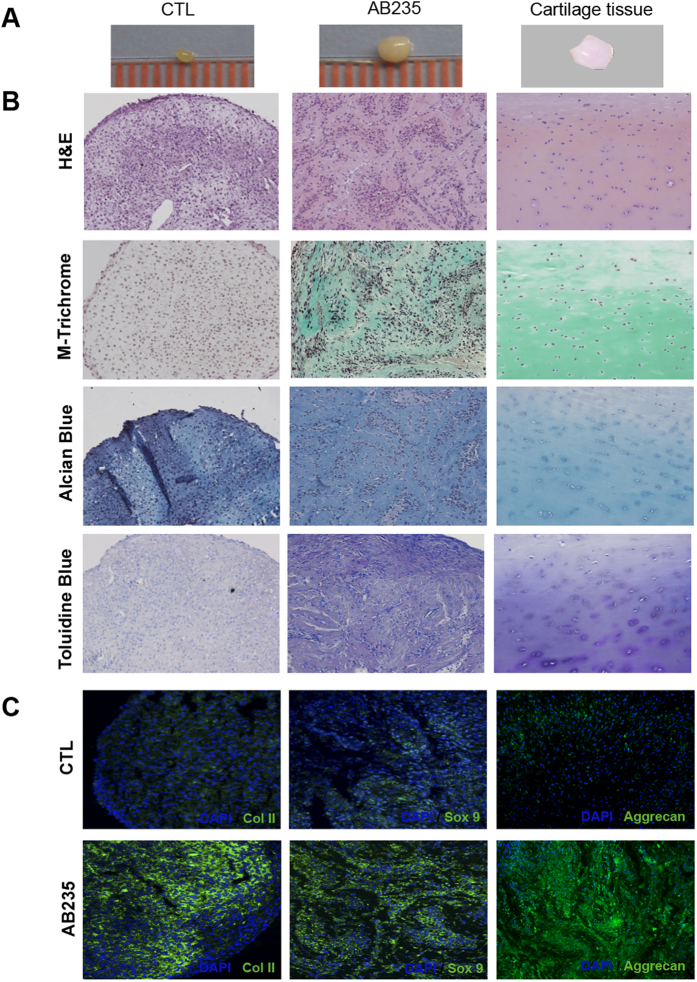
AB235 induces chondrocyte redifferentation *in vitro*. (**A**) Representative images of dedifferentiated chondrocytes cultured in a pellet system for 6 weeks in the absence of treatment (CTL) or treated with 10 ng/ml of the chimeric ligand (AB235) are compared with an image of native cartilage tissue. (**B**) Histological staining of sections of the pellets from **(A)** shows the acquisition of a cartilage like matrix resulting from AB235 treatment. (**C**) Merged images of pellet sections inmunostained with Col II, Sox9 and Aggrecan antibodies (green channel) and cell nuclei labelled with DAPI (blue channel) demonstrate that AB235 treatment increases of chondrogenic marker expression. Original magnification: 20× for all panels.

We tested if the chimeric AB235 ligand could induce redifferentiation of chondrocytes that have lost their differentiated phenotype following extended monolayer culture. Dedifferentiated cells were cultured for 4 weeks under pellet-forming conditions in chondrogenic medium either containing or lacking the AB235 chimeric ligand and then analyzed using histological and immunofluorescence probes. Pellets cultured in AB235-containing medium showed a noticeable increase in size relative to untreated controls. In addition, the AB235-induced pellets had a consistency and appearance more similar to native cartilage tissue Fig. 2(A). We compared the internal structure of the AB235-treated and non-treated pellets and found that the ECM of treated pellets was also more similar to that of native cartilage tissue Fig. 2(B). Pellet sections stained for H&E showed that AB235 induced the formation of a complex cellular organization with cells embedded in lacunae surrounded by ECM (staining in pale pink), again resembling native cartilage tissue, while ECM was not visible when cells were cultured under control conditions. Masson-Trichrome staining revealed collagen-specific staining (green) in the ECM of AB235-treated pellet sections that was similar to the staining of the native tissue but not visible in control pellets. Cartilage specific proteoglycans were also clearly more apparent in AB235-treated pellets than in the control as assessed by Alcian Blue and Toluidine Blue assays pellets (blue and purple respectively).

Further, Col II, Sox 9 and Aggrecan were expressed in the AB235-induced pellets at significantly higher levels than in the control pellets that showed weak and diffuse staining for these markers of chondrocyte differentiation Fig. 2(C). The patterned arrangement of Col II and Aggrecan fibres is striking and in stark contrast with the homogeneous distribution of this protein in control pellet sections.

**Figure 3:**
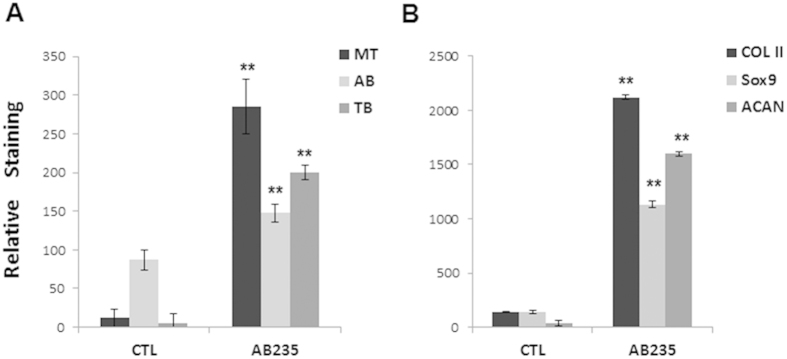
Quantitative image analysis. Graphical representation of the quantification of histological (**A**) and immunofluorescence (**B**) staining of control and AB235-treated pellet sections. MT: Masson-Trichrome; AB: Alcian Blue; TB: Toluidine Blue; ACAN: Aggrecan. **Statistical significance indicated (p < 0.01).

Finally, quantitative image analysis was performed using ImageJ. For histological and immufluorescence images, the staining of AB235-treated pellets was significantly higher (p < 0.01) in all cases when compared with control pellets Fig 3.Together, these data indicate that AB235 promotes re-establishment of the chondrocytic phenotype in cells that lose this phenotype following extended culture periods *in vitro*.

### Chondrocytes redifferentiated by AB235 *in vitro* promote cartilage integration upon transplantation in mice

**Figure 4:**
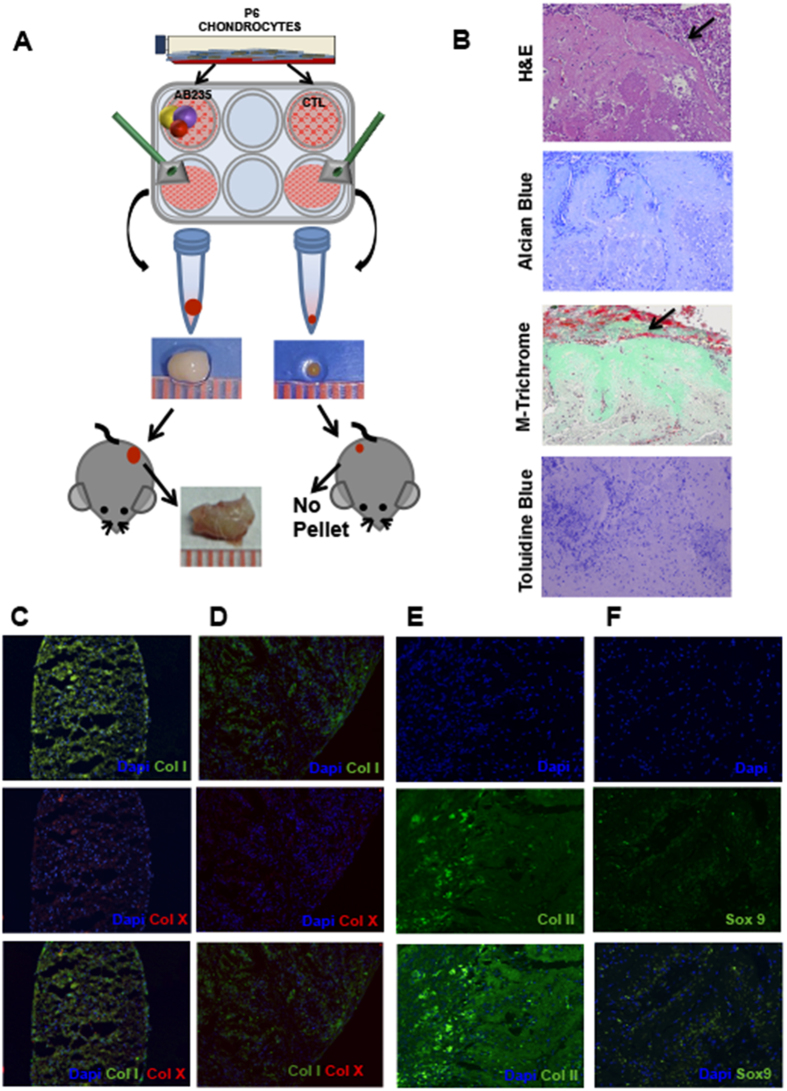
AB235 induces chondrocyte redifferentiation *in vivo*. (**A**) Schematic representation of the experimental design showing images of representative pellets before and after implantation into mice and integration of AB235-treated pellets with the surrounding tissue. (**B**) Sections of AB235-treated pellets harvested from mice and stained for H&E, Masson’s Trichrome, Alcian Blue and Toluidine Blue show a robust staining for mature, cartilage-like ECM. Black arrows indicate the edge of the pellet in H&E and Masson’s Trichrome stained sections while the edge of the pellet is not visible in Alcian Blue and Toluidine Blue stained sections. **(C–F)** Representative images of immunofluorescence analysis of cartilage markers. Stained sections of fibrotic marker type I collagen (Col I) and hypertrophic marker type X collagen (Col X) in both control pellet grown *in vitro* (**C**) and AB235-induced pellet harvested from mice (**D**). Expression of the chondrogenic markers Col II and Sox 9 in AB235 induced pellet sections after the *in vivo* assay (**E**,**F**). Original magnification 10× for (**C,D**); 20× for (**E**,**F)**.

We tested whether chondrocytes redifferentiated as a pellet *in vitro* are capable of maintaining their 3D structure after being transplanted into mice. Figure 4A shows a schematic representation of the experimental design we employed. Pellets obtained after 6 weeks of culture in the presence or absence of AB235 were transplanted into subcutaneous tissue on the flanks of inmunodeficient mice and then harvested 4 weeks later for histological and immunofluorescence analysis.

We find that the control pellets are completely absorbed by the surrounding mouse tissue and could not be recovered for histological and immunofluorescence analysis. By contrast, AB235-treated pellets displayed a dramatic increase in size over the 4 week period demonstrating that the graft was well tolerated by the organism Fig. 4(A). Histological analysis reveals that the ECM synthesized by redifferentiated chondrocytes is cartilage-specific pericellular matrix consisting primarily of vertically-oriented collagen fibres and proteoglycans Fig. 4(B). Integration of the AB235-treated pellet into the surrounding mouse tissue is demonstrated by the formation of *novo* tissue around the pellet that can be seen in the H&E stained section as shown in Fig. 4B. Finally, our immunofluorescence assay for collagens I and X shows that AB235 treatment does not induce fibrotic or hypertrophic cartilage formation Fig. 4(C,D). On the other hand, collagen II and Sox 9 markers were highly expressed with an arranged Col II distribution typical of a structured ECM and with Sox 9 localized in both the nucleus and cytoplasm Fig. 4(E,F). Finally, our results showed that Col X was almost undetectable in AB235-treated cells.

## Discussion

Autologous chondrocytes are suitable for cell therapy strategies directed to repair cartilage tissue degeneration or damage. However, these strategies are hampered by the fact that chondrocytes undergo dedifferentiation when they are grown in monolayer culture for prolonged periods^2^. To overcome this limitation, we developed a robust protocol to redifferentiate chondrocytes that have undergone such *in vitro* culture-induced dedifferentiation.

Chondrocyte dedifferentiation has been described to occur as soon as 4–10 days after cells are plated in a monolayer^4^. We ensured full dedifferentiation toward a fibroblastic phenotype by growing chondrocytes in monolayer culture over a period of 4 weeks. Morphological, histological and immunological analysis together with real-time PCR measurement of Col I and Col II gene expression confirmed the complete dedifferentiation of chondrocytes over this 4 week period in a manner that is in agreement with prior findings^13^.

We have previously reported the creation of chimeric ligands that combine BMP2 and Activin-A sequences^10^. We subsequently confirmed the chondrogenic potential of Activin- and Nodal-like chimeras including AB235 in directing chondrogenic differentiation of adipose derived stem cells^11^,^14^. In the present study, we specifically hypothesized that AB235 reverts dedifferentiated chondrocytes back to their previous, fully differentiated chondrocytic state. We tested this hypothesis by culturing dedifferentiated chondrocytes under 3D conditions. It is known that cell-to-cell contact promotes chondrogenic differentiation and 3D culturing has been used before to induce redifferentiation of monolayer-expanded autologous chondrocytes^5^. However, our results showed that 3D culturing is not sufficient *per se* to redifferentiate chondrocytes as proven by the small size and lack of proper tissue organization found in the control pellets. On the other hand, pellets cultured with AB235 produced a cartilaginous matrix comparable to the ECM found in native cartilage tissue. These findings suggest that the AB235 ligand interacts with Activin/BMP receptors in a way that promotes the reversion toward a chondrocytic phenotype in a manner concordant with what previous studies using BMP2 have shown^15^,^16^. In fact, the enhanced chondrogenic differentiation of human adipose derived stem cells (hASCs) treated with AB235 relative to that of cells treated with BMP2 suggests that this chimeric ligand signals more efficiently than BMP2 through type I and type II receptors that mediate cartilage maturation^11^.

Furthermore, we demonstrate that AB235-induced cartilage integrates into the subcutaneous tissue upon transplantation into the flanks of immune compromised mice and that the structural cartilage-like complexity of the ECM in AB235-treated pellets strongly resembles native cartilage. By contrast, non-treated control pellets were absorbed by the surrounding tissue and could not be recovered for examination, in agreement with others studies that have showed that dedifferentiated chondrocytes failed to form cartilage tissue *in vivo*^17^,^18^. Interestingly, Activin A has been shown to be an inhibitor of matrix metalloproteinase 3 and to block the degradation of the ECM by this enzyme^19^ raising the possibility that AB235, which utilizes the activin pathway, may exert similar effects.

From the present study we can conclude that the combination of sequences of BMP2 and Activin-A present in AB235 result in a ligand that increases the expression of chondrogenic markers of dedifferentiated chondrocytes. Others studies have shown that BMP2 upregulates chondrogenic gene expression of human articular chondrocytes expanded *in vitro*^20^ and, therefore, comparing the chondrogenic potential of BMP2 versus AB235 would be of great interest for future studies.

In conclusion, we describe an effective protocol for redifferentiation of autologous chondrocytes obtained from OA patients and the formation of a cartilage-like ECM that can integrate into the surrounding tissue *in vivo*. Future work will include assessment of the tumour-forming potential of AB235-treated cells in order to determine if the procedure can be translate to the clinic. Since the success of cell therapies for cartilage injury depends on the quality and quantity of the implanted cells, our protocol may have significant potential for clinical applications.

## Material and Methods

### Patients

Articular cartilage was obtained from from 4 female and 4 male patients with knee osteoarthritis during joint replacement surgery at the University Hospital of Málaga, Spain, according to the guidelines of the University Hospital of Malaga (Table S1). Informed patient consent was obtained for all samples used in this study and samples were collected in accordance with the Research Ethics Committees of the University Hospital of Malaga, “Virgen de la Victoria” and University of Granada (ES180870000164). Average patient age at resection for females and males was 67,25 ± 7 years; and 66,5 ± 6 years, respectively. None of the patients had a history of inflammatory arthritis or crystal-induced arthritis. Human articular cartilage (leaf shape; size: length: 16 ± 5 mm and width: 8 ± 2 mm) was obtained from the femoral side, selecting the non-overload compartment: lateral condyle in varus deformity and medial condyle in valgus cases. Only cartilage that looked normal microscopically was used for this study. Samples collected at joint arthroplasty were transported to the laboratory in Dulbecco’s modified Eagle’s medium (DMEM; Sigma) with 100 U/ml penicillin and 100 mg/ml streptomycin.

### Isolation and dedifferentitation of human articular chondrocytes

Articular chondrocytes were isolated as previously described^12^. To induce chondrocyte dedifferentiation cells were harvesting by TrypLE (Invitrogen) and cultured on monolayer for 6–7 passages.

### Chondrogenic differentiation in cell pellet culture

Chondrocytes were maintained at 37 °C in a humidified atmosphere containing 20% O2 and 5% CO2 usin a protocol detailed in Supplementary data. Briefly, 200,000 cells/ml were grown using a pellet system as described before^11^. Control pellets were grown in DMEM–high glucose (Sigma) Supplemented with 10% foetal bovine serum (FBS, Gibco, composition is listed in supplementary data), 50 μg/μL of l-ascorbic acid 2-phosphate (Sigma), 1% penicillin-streptomycin (Sigma) and 1% ITS (Insulin-Transferrin-Selenium, Gibco). To induce redifferentiation, 10 ng/ml of AB235 was added fresh during each media exchange every 48 hours. The AB235 chimeric ligand was generated as previously described^10^ and was dissolved in a solution (1:20 v/v) of 10 mM sodium acetate buffer (pH 5.0) and PBS containing 0,1% bovine serum albumin (BSA).

### RNA isolation and real time-PCR analysis

Total cellular RNA isolation, cDNA generation and Real-time PCR were performed as described in Supplementary data. Primer sequences used in this study are summarized in Table S1 (Supplementary data).

### Histological and immunohistochemical analysis

Described in detail in Supplementary data.

### *In vivo* assay

*In vivo* experiments were performed in immunodeficient NOD SCID (NOD.CB17-Prkdc^scid^/NcrCrl) mice purchased from Charles River (Barcelona, Spain). Cell pellets obtained after 6 weeks of chondrogenic induction were transplanted into the back subcutaneous tissue of mice anesthetized (n = 6) by isoflurane inhalation (described in detail in Supplementary data). *In vivo* assays were carried out in accordance with the approved guidelines of University of Granada following institutional and international standards for animal welfare and experimental procedure. All experimental protocols were approved by the Research Ethics Committee of the University of Granada.

### Statistical analysis

All graphed data represent the mean + /-SD from at least three experiments. Differences between treatments were tested using the two tailed Student´s T test. Assumptions of Student´s T test (homocedasticity and normality) were tested and assured by using transformed data sets [log(dependent variable value + 1)] when necessary. P-values < 0.01 (**) were considered statistically significant in all cases.

## Additional Information

**How to cite this article**: Jiménez, G. *et al*. Activin A/BMP2 chimera AB235 drives efficient redifferentiation of long term cultured autologous chondrocytes. *Sci. Rep*. **5**, 16400; doi: 10.1038/srep16400 (2015).

## Supplementary Material

Supplementary Information
