# Supplementary material for: Activin A/BMP2 chimera AB235 drives efficient redifferentiation of long term cultured autologous chondrocytes
Source: Sci Rep. 2015 Nov 13;5:16400. doi: 10.1038/srep16400 (PMC4643338; doi:10.1038/srep16400)
Supplement: Supplementary Information [file srep16400-s1.pdf]

Activin A/BMP2 chimera AB235 drives efficient redifferentiation of long term cultured autologous chondrocytes

G. Jiménez, E. López-Ruiz, W. Kwiatkowski, E. Montañez, F. Arrebola, E. Carrillo, P.C. Gray, J.C. Izpisua Belmonte, S. Choe, M. Perán, J. A. Marchal.

Material and Methods

RNA isolation and real time-PCR analysis

Total cellular RNA was isolated using TriReagent (Sigma) and reverse transcribed using the Reverse Transcription System kit (Promega). Real-time PCR was performed using the SYBR-Green PCR Master mix (Promega) according to the manufacturer's recommendations. PCR reactions were performed as follows: an initial denaturation at 95°C for 2 min, 40 cycles of 95°C for 5 s and 60°C for 30 s, and final cycle of dissociation of 60 – 95 °C. The gene expression levels were normalized to corresponding GAPDH values and are shown as fold change relative to the value of the control sample. All the samples were done in triplicate for each gene.

Histological analysis

Cartilage tissue and cell pellets were immersed in 4% paraformaldehyde in 0.1 M phosphate buffered saline (PBS) for 4 hours at 4°C, washed in 0.1M PBS and embedded in paraffin in an automatic tissue processor (TP1020, Leica, Germany). The paraffin blocks were cut into 4 µm sections for staining. Alsian blue and Toluidine blue reveal

the presence of glycosaminoglycans (blue and purple respectively), and Masson-trichrome shows the existence of collagens (green).

Immunohistochemical analysis

Fixed section and monolayer were blocked for 1 hour at room temperature (RT) with 5% BSA, 5% foetal bovine serum in PBS and then incubated with Col I (SC25974, St. Cruz), Col X (ab49945, Abcam); Col II (SC52658, St. Cruz) and Sox 9 (AB5535, Millipore) overnight at 4°C. The next day, samples were washed thrice with PBS and incubated with the secondary antibodies (St. Cruz) for 1 hour at RT and finally, were washed thrice with PBS and mounted with mounting medium with DAPI.

Quantitative image analysis

Quantitative image analysis was performed using ImageJ software (v1.43, NIH). Area, integrated density and mean staining were measured, along with several adjacent background readings. The relative staining was calculated using = integrated density – (selected area × mean staining of background readings)[1,2]. All the samples were done in triplicate, different sections of the pellets were analysed.

Chondrogenic differentiation in cell pellet culture

Chondrocytes were cultured during 6-7 passages to adchieve full differentiation. 200.000 cells/ml of differentiated chondrocytes were plated into a well of six well plate. Control cells were grown in incomplete chondrogenic medium (DMEM–high glucose supplemented with 10% foetal bovine serum, 50 µg/µL of l-ascorbic acid 2-phosphate,

1% penicillin-streptomycin and 1% ITS. Treated cells were cultured in incomplete chondrogenic medium supplemented with 10 ng/ml of AB235. Media was change every other day and fresh AB235 was added to treated pellet during each medium change. After two weeks in culture, control and treated monolayer cells were manually separated using a sterile scraped, transferred to 15 ml conical tubes and centrifuged at 300 g at 21 °C for 7 minutes to form a cell pellet. Control and treated pellets were incubated for four weeks, medium was changed every other day and fresh AB235 was added to treated pellet during each medium change.

In vivo assay

Animals were maintained in a microventilated cage system with a 12-h light/dark cycle with food and water ad libitum. Mice (n=6) were manipulated in a laminar air-flow to keep on the specific pathogen-free conditions. Cells were grown on pellet system as described before[8], and after 6 weeks 2 AB235 pellets and 2 control pellets were obtained from each of the 3 different patients. 2 pellets (AB235 and control) of the same patient were implanted into the back subcutaneous tissue of anesthetized mice (n=2), one on each side of the midline. Four weeks later, the mice were sacrificed via an overdose injection of anaesthetic, and the pellet with the new tissue formed around it were excised for further histologic analysis. Animal welfare and experimental procedures were carried out in accordance with institutional and international standards.

Table S1. Patient data and evaluation of the conditions of the knee according the Ahlback scale value and the Knee Society Knee Scoring System (KSS)

Patient	Sex (M/F)	Age (Years)	Ahlback	KSS
1	M	66	III	30
2	F	74	III	28
3	M	67	II	32
4	M	74	III	25
5	M	59	III	37
6	F	70	III	29
7	F	68	III	30
8	F	57	II	35

Table S2. Sequences of the primers used in the RT-PCR reactions

Gene	Forward	Reverse
Collagen I	ATGGATGAGGAAACTGGCAACT	GCCATCGACAAGAACAGTGTAAGT
Collagen II	GAGACAGCATGACGCCGAG	GCGGATGCTCTCAATCTGGT
Collagen X	GCC CAC TAC CCA ACA C	TGG TTT CCC TAC AGC TGA
Sox 9	ACTCCGAGACGTGGACATC	TGTAGGTGACCTGGCCGTG
GADPH	TGCACCACCAACTGCTTAGC	GGCATGGACTGTGGTCATGAG

Table S3. Compossition of the Foetal Bovine Serum used in this study. Provided by Technical Application Scientist II. Life Sciences Solutions. Thermo Fisher Scientific

Biochemical Component	Units	Fetal Bovine Serum mean	(range)
Total Protein	g/dl	4,2	4.0 - 4.3
pH	units	7,3	6.7 - 7.3
Osmolality	mosm/kg	314	290 - 335
Glucose	mg/dl	130	107 - 144
Hemoglobin	mg/dl	14,2	5.8 - 23.0
Bilirubin	mg/dl	0,2	0.1 - 0.4
Uric Acid	mg/dl	3,2	2.6 - 3.5
Urea Nitrogen	mg/dl	14,4	15 - 18
Creatinine	mg/dl	3,1	2.8 - 3.3
Sodium	meq/L	134	131 - 137
Potassium	meq/L	15,1	12.9 - 14.2
Calcium (total)	mg/dl	14,6	14.3 - 15.0
Chloride	meq/L	104,9	102 - 108
Phosphorus (inorganic)	mg/dl	11,2	10.0 - 14.0
Iron (total)	ug/dl	195	189 - 204
Albumin	g/dl	2,6	1.3 - 2.9
Globulin (total)	g/dl	1,3	1.1- 1.5
Alkaline Phosphatase	U/L	262	2 - 319
GG-Transpeptidase	U/L	5.0	0 - 7.0
SGOT	U/L	60.4	28 - 161
Lactate Dehydrogenase	U/L	559	262 - 1010
Cholesterol	mg/dl	34.2	19 - 54
Low Density Lipoprotein	mg/dl	2.8	0 - 7.0
High Density Lipoprotein	mg/dl	6.5	5.0 - 9.0
Triglycerides	mg/dl	219.5	73- 1720
Growth Hormone	ng/ml	131	126 - 138
Insulin	uIU/ml	4.3	2.9 - 5.5
Estradiol	pg/ml	13.8	11.2 - 17.5
Progesterone	ng/ml	0.03	0.01 - 0.06
Testosterone	ng/ml	0.40	0.38 - 0.45
T4 (Thyroxine)	ug/dl	14.8	13.9 - 15.8
T3	ng/ml	1.2	0.9 - 1.4

Supplementary References

1. Jensen, E.C. Quantitative analysis of histological staining and fluorescence using ImageJ. *Anat Rec.* **296(3),** 378-381 (2013).

2. McCloy, R.A. *et al*. Partial inhibition of Cdk1 in G2 phase overrides the SAC and decouples mitotic events. *Cell Cycle*. **13(9),** 1400-1412 (2014).
